# Comprehensive information-based differential gene regulatory networks analysis (CIdrgn): Application to gastric cancer and chemotherapy-responsive gene network identification

**DOI:** 10.1371/journal.pone.0286044

**Published:** 2023-08-23

**Authors:** Heewon Park, Seiya Imoto, Satoru Miyano

**Affiliations:** 1 School of Mathematics, Statistics and Data Science, Sungshin Women’s University, Seoul, Korea; 2 Human Genome Center, The Institute of Medical Science, The University of Tokyo, Minato-ku, Tokyo, Japan; 3 M&D Data Science Center, Tokyo Medical and Dental University, Bunkyo-ku, Tokyo, Japan; Anadolu University: Anadolu Universitesi, TURKEY

## Abstract

Biological condition-responsive gene network analysis has attracted considerable research attention because of its ability to identify pathways or gene modules involved in the underlying mechanisms of diseases. Although many condition-specific gene network identification methods have been developed, they are based on partial or incomplete gene regulatory network information, with most studies only considering the differential expression levels or correlations among genes. However, a single gene-based analysis cannot effectively identify the molecular interactions involved in the mechanisms underlying diseases, which reflect perturbations in specific molecular network functions rather than disorders of a single gene. To comprehensively identify differentially regulated gene networks, we propose a novel computational strategy called comprehensive analysis of differential gene regulatory networks (CIdrgn). Our strategy incorporates comprehensive information on the networks between genes, including the expression levels, edge structures and regulatory effects, to measure the dissimilarity among networks. We extended the proposed CIdrgn to cell line characteristic-specific gene network analysis. Monte Carlo simulations showed the effectiveness of CIdrgn for identifying differentially regulated gene networks with different network structures and scales. Moreover, condition-responsive network identification in cell line characteristic-specific gene network analyses was verified. We applied CIdrgn to identify gastric cancer and itsf chemotherapy (capecitabine and oxaliplatin) -responsive network based on the Cancer Dependency Map. The CXC family of chemokines and cadherin gene family networks were identified as gastric cancer-specific gene regulatory networks, which was verified through a literature survey. The networks of the olfactory receptor family with the ASCL1/FOS family were identified as capecitabine- and oxaliplatin sensitive -specific gene networks. We expect that the proposed CIdrgn method will be a useful tool for identifying crucial molecular interactions involved in the specific biological conditions of cancer cell lines, such as the cancer stage or acquired anticancer drug resistance.

## Introduction

The complex mechanisms underlying some diseases states cannot be understood by gene expression level analyses alone because some diseases are caused by disturbances in the specific functions of molecular networks rather than disorders in a single gene. One of the most powerful emerging techniques for biomedical research is heterogeneous gene regulatory network analysis, especially for responsive gene sets and module identification, and such networks have attracted considerable research attention because of their ability to reveal the complex mechanisms of diseases. Many studies have focused on the expression levels of genes and developed statistical methods for identifying differentially expressed genes (DEGs) or gene sets [1–3]. Computational methods based on correlations between genes have also been proposed to identify condition-responsive subnetworks and gene modules. Tesson et al. [4] focused only on the coexpression of genes and developed the method DiffCoEx to identify correlation pattern changes between multiple conditions. Guo et al. [5] developed the responsive score based on covariance of genes and then proposed the method for searching responsive networks. Although various computational approaches have been developed to identify significant gene sets describing phenotype-specific characteristics, these methods are based only on partial or incomplete information (e.g., correlation, covariance of expression levels of genes) on gene regulatory networks. Thus, biological condition-responsive gene networks could not be effectively identified.

Our study focuses on the drawbacks of the previous studies. In order to consider various aspects of network structure and effectively identify responsive networks, we propose a novel computational strategy that incorporates comprehensive information of network structure unlike the existing studies, called comprehensive information-based differential gene regulatory network analysis (CIdrgn). We consider the gene regulatory network structure based on not only expression levels of genes but also edge structures and regulatory effects of regulator genes to their target genes and incorporate the comprehensive information into a dissimilarity measure to compare the gene regulatory networks. We then proposed a novel statistic strategy to identify differentially regulated gene networks across phenotypes or biological conditions and extend the proposed CIdrgn to cell line characteristic-specific gene regulatory network analysis. To the best of our knowledge, this is the first study on responsive network identification based on comprehensive information of network structure.

We demonstrate the effectiveness of the proposed CIdrgn scheme through Monte Carlo simulations. Our strategy showed outstanding performance for identifying differentially regulated gene networks with various structures and scales. We applied our strategy to the Cancer Dependency Map (DepMap) dataset and performed gastric cancer (GC) and chemotherapy (capecitabine and oxaliplatin)-responsive gene network analyses. The identified responsive networks and their members (genes) represent candidate biomarkers for mechanisms related to gastric/colorectal cancer and various chemotherapies. Although biological knowledge about the identified subnetworks and markers was not incorporated into the differential gene regulatory network analysis, our data-driven strategy provides biologically reliable results for GC and its chemotherapy-responsive gene network analysis. Our results suggested that The CXC family of chemokines and cadherin (CDH) genes may play crucial roles in tumor invasion, metastasis, and progression of GC. In addition, the loss of activity of the olfactory receptor (OR) family with the ASCL1/FOS family may lead to drug resistance in cell lines and inducing these gene families may provide vital clues to enhance the chemotherapy efficiency of capecitabine and oxaliplatin.

The remainder of this paper is organized as follows. In the Methods section, we introduce a novel strategy for the differential gene regulatory network analysis. In the Monte Carlo simulation section, we present the results of the simulation studies. In the GC and XELOX-responsive gene regulatory network analysis section, we describe the results of GC and its chemotherapy-responsive network analysis. In the Discussion section, we provide the conclusions.

## Methods

Suppose that $X={\left(x_{1},\ldots ,x_{n}\right)}^{T}\in R^{n\times p}$ is an *n* × *p* data matrix that describes the expression of *p* possible regulators that control target gene transcription $y_{\ell }\in R^{n},\ell =1,\ldots ,q$ for *n* cell lines. To estimate the gene regulatory network, we considered the following linear regression model:

$$\begin{array}{c} y_{\ell }=\sum_{j=1}^{p}\beta_{\ell j}x_{j}+\epsilon_{\ell },\;\ell =1,\ldots ,q, \end{array}$$ (1)

where *β*_*ℓj*_ is the regression coefficient that represents the effect of each regulator ***x***_*j*_ on its target ***y***_*ℓ*_ and ***ϵ***_*ℓ*_ = (*ϵ*_*ℓ*1_, …, *ϵ*_*ℓn*_)^*T*^ is a random error vector. The network can be represented by a weighted graph *G* = (*V*, *E*, *W*), where *V* is the set of vertices corresponding to *p* genes and *E* ∈ *V* × *V* is the set of edges, where (*i*, *j*) ∈ *E* indicates a link between vertices *i* and *j* (i.e., genes *i* and *j*). *W* = (*w*_*ij*_) is the edge weight between vertices *i* and *j* which can be estimated using ${\hat{\beta }}_{ij}$ and ${\hat{\beta }}_{ji}$.

### Previous studies

#### Significance analysis of microarray for gene sets (SAM-GS)

To identify statistically significant gene sets across phenotypes A and B, Dinu et al. [1] proposed the SAM-GS method by extending the SAM t-statistic. The SAM-GS statistic is given as follows:

$$\begin{array}{c} D_{\text{SAM-GS}}=\sum_{j\in V}\frac{{\left({\bar{x}}_{Aj}-{\bar{x}}_{Bj}\right)}^{2}}{s_{j}+s_{0}}, \end{array}$$ (2)

where ${\bar{x}}_{Aj}$ and ${\bar{x}}_{Bj}$ are the averages of the expression levels of gene *j* in phenotypes A and B, respectively; *s*_0_ is a tuning parameter; and *s*_*j*_ is the gene-specific scatter. This scatter is given as follows:

$$\begin{array}{c} s\left(j\right)=\sqrt{a\{\sum_{i=1}^{n_{A}}{\left(x_{ij}-{\bar{x}}_{Aj}\right)}^{2}+\sum_{k=1}^{n_{B}}{\left(x_{kj}-{\bar{x}}_{Bj}\right)}^{2}\}}, \end{array}$$ (3)

where *n*_*A*_ and *n*_*B*_ are the numbers of cell lines in phenotypes A and B, respectively, and *a* = (1/*n*_*A*_ + 1/*n*_*B*_)/(*n*_*A*_ + *n*_*B*_ − 2). The SAM-GS statistic measures the difference in expression levels of a gene set across phenotypes A and B.

#### Gene set co-expression analysis (GSCA)

Choi and Kendziorski [6] proposed GSCA a statistical method to identify differentially co-expressed genes. For genes in a subnetwork *G*, GSCA computes pairwise correlations for all (|V|2) gene pairs, where |*V*| is the number of genes in *V*. The statistic of the GSCA used to measure the dispersion of correlations between phenotypes A and B is given as follows:

$$\begin{array}{c} D_{\text{GSCA}}=\sqrt{\frac{1}{|V|(|V|-1)/2}\sum_{k=2}^{\left|V\right|}\sum_{j=1}^{k-1}{\left(c_{kj}^{A}-c_{kj}^{B}\right)}^{2}}, \end{array}$$ (4)

where $c_{kj}^{A}$ and $c_{kj}^{B}$ are the correlations between the *k*^*th*^ and *j*^*th*^ genes for the phenotypes *A* and *B*, respectively.

### Comprehensive information-based differentially regulated gene network analysis

In this study, we proposed a novel differential gene regulatory network analysis strategy that can effectively identify biological condition-responsive subnetworks based on gene expression levels and subnetwork structures. Network structures are evaluated based on regulatory effects between genes and the edge structure of genes, and the following statistics are proposed to measure the dissimilarity of the subnetworks.

- Dissimilarity based on regulatory effects between genes
  We define the following regulatory effect of the *j*^*th*^ regulator gene on the *ℓ*^*th*^ target gene in phenotypes A and B, consistent with a study conducted earlier [7]:
  $$\begin{array}{c} r_{\ell j}\left(A\right)={\hat{\beta }}_{A\ell j}{\bar{x}}_{Aj}\;\text{and}\;r_{\ell j}\left(B\right)={\hat{\beta }}_{B\ell j}{\bar{x}}_{Bj}\;\text{for}\;j=1,\ldots ,p,\;\ell =1,\ldots ,q, \end{array}$$ (5)
  where ${\hat{\beta }}_{A\ell j}$ and ${\hat{\beta }}_{B\ell j}$ are the regression coefficients estimated by cell lines of phenotypes of A and B, respectively. Let ***R***(*A*) = {*r*_1*j*_(*A*), …, *r*_*qj*_(*A*), *r*_*j*1_(*A*), …, *r*_*jp*_(*A*)}^*T*^ and ***R***(*B*) = {*r*_1*j*_(*B*), …, *r*_*qj*_(*B*), *r*_*j*1_(*B*), …, *r*_*jp*_(*B*)}^*T*^ be regulatory effect vectors of the *j*^*th*^ gene in phenotypes A and B, respectively, where the first *q* elements indicate the regulator effects of *j*^*th*^ gene on its *q* targets and the remaining *p* elements indicate the regulator effect of the *p* regulators on the *j*^*th*^ gene. We propose the following statistic to describe dissimilarity of the regulatory effect of the *j*^*th*^ gene:
  $$\begin{array}{c} \gamma_{j}\left(A,B\right)=\frac{1}{q+p}||R\left(A\right)-{R\left(B\right)||}_{2}^{2}. \end{array}$$ (6)
  The large value *γ*_*j*_(*A*, *B*) indicates the *j*^*th*^ gene has different expression levels and size of edges connected its neighborhoods between phenotypes A and B. We then propose the following statistic to measure dissimilarity of regulatory effects of a subnetwork *G*:
  $$\begin{array}{c} \Gamma =\frac{1}{\left|V\right|}\sum_{j\in V}\gamma_{j}\left(A,B\right). \end{array}$$ (7)
  The large value of Γ indicates that the genes consisting of *G* have different regulatory effects on their target genes across the phenotypes, thus the network corresponding the large Γ can be considered as the responsive network having phenotype specific regulatory effects.
- Dissimilarity of the edge structure of nodes
  The genes linked in the networks may have similar biological functions; thus, node (gene) similarity can be defined using the Jaccard index. We propose the following statistic for the node similarity of the *j*^*th*^ gene between phenotypes A and B in line with Li et al. [8]:
  $$\begin{array}{c} N_{j}\left(A,B\right)=\frac{|N_{Aj}\cap N_{Bj}|}{|N_{Aj}\cup N_{Bj}|}, \end{array}$$ (8)
  where *N*_*Aj*_ and *N*_*Aj*_ are the sets of nodes that are directly connected to the *j*^*th*^ gene within phenotypes A and B, respectively. If the gene have similar target and regulator genes between phenotypes A and B, than we consider that the gene is not phenotype specific feature. Thus, we propose the following Jaccard distance metric to measure the dissimilarity of the edge structure of the *j*^*th*^ gene:
  $$\begin{array}{c} \lambda_{j}\left(A,B\right)=1-N_{j}\left(A,B\right). \end{array}$$ (9)
  We then propose the following statistic to describe the dissimilarity of edge structures in a subnetwork:
  $$\begin{array}{c} \Lambda =\frac{1}{\left|V\right|}\sum_{j\in V}\lambda_{j}\left(A,B\right). \end{array}$$ (10)
- Dissimilarity based on adjusted regulatory effects
  We also consider the following statistic based on the adjusted regulatory effect using the Jaccard distance:
  $$\begin{array}{c} \Gamma_{\Lambda }=\frac{1}{\left|V\right|}\sum_{j\in V}\{\gamma_{j}\left(A,B\right)+\gamma_{j}\left(A,B\right)\cdot \lambda_{j}\left(A,B\right)\}. \end{array}$$ (11)

To comprehensively measure the dissimilarity of a subnetwork across biological conditions or phenotypes, we propose the following statistic that incorporates not only expression levels of genes but also edge structures and regulatory effects into the differential gene regulatory network analysis,

$$\begin{array}{cc} & \text{CIdrgn}.1=\Gamma_{\Lambda }+D_{\text{SAM}-\text{GS}}, \end{array}$$ (12)

$$\begin{array}{cc} & \text{CIdrgn}.2=\Gamma +\Lambda +D_{\text{SAM}-\text{GS}}. \end{array}$$ (13)

The large values of the statistics CIdrgn.1 and CIdrgn.2 indicate that the subnetworks show different regulatory structures for phenotypes A and B.

#### Responsive subnetwork extract

To extract biological condition-responsive subnetworks, we computed the significance of the proposed statistics based on the permutation framework.

We first compute the statistics Γ, Λ, and Γ_Λ_ for the subnetworks. Then, we generate permutation samples $S_{A}^{\left(pm\right)}$ and $S_{B}^{\left(pm\right)}$ and estimate gene regulatory networks *G*^(*pm*)^(*A*) and *G*^(*pm*)^(*B*) based on $S_{A}^{\left(pm\right)}$ and $S_{B}^{\left(pm\right)}$ for *pm* = 1, …, *T*. The permutation statistics Γ^(*pm*)^, Λ^(*pm*)^, and $\Gamma_{\Lambda }^{\left(pm\right)}$ were computed for *G*^(*pm*)^(*A*) and *G*^(*pm*)^(*B*). Because the statistics have different scales, we normalize each statistic **Γ**, **Λ**, and **Γ**_Λ_ to prevent that any one dominates CIdrgn.1 and CIdrgn.2, where

$$\begin{array}{c} \Gamma ={\left(\Gamma ,\Gamma^{\left(1\right)},\ldots \Gamma^{\left(T\right)}\right)}^{T},\\ \Lambda ={\left(\Lambda ,\Lambda^{\left(1\right)},\ldots \Lambda^{\left(T\right)}\right)}^{T},\\ \Gamma_{\Lambda }={\left(\Gamma_{\Lambda },\Gamma_{\Lambda }^{\left(1\right)},\ldots \Gamma_{\Lambda }^{\left(T\right)}\right)}^{T}. \end{array}$$ (14)

For notational compactness, in the remainder of this section, we shall write the notation in Eq (14) as normalized statistics.

We then computed the similarity measures CIdrgn.1 and CIdrgn.2 based on the normalized statistics and computed the following permutation p-values:

$$\begin{array}{c} p.{\text{value}}_{\text{CIdrgn}.1}=\frac{\sum_{pm=1}^{T}I(\left\{\text{CIdrgn}.1\leq {\text{CIdrgn}}^{\left(pm\right)}.1\right)}{T}, \end{array}$$ (15)

$$\begin{array}{c} p.{\text{value}}_{\text{CIdrgn}.2}=\frac{\sum_{pm=1}^{T}I\left(\left\{\text{CIdrgn}.2\leq {\text{CIdrgn}}^{\left(pm\right)}.2\right\}\right)}{T}, \end{array}$$ (16)

where CIdrgn^(*pm*)^.1 and CIdrgn^(*pm*)^.2 are the statistics computed by the *pm*^*th*^ permutation sample and ***I***(⋅) is an indicator function. The small permutation p-value indicates that networks *G*(*A*) and *G*(*B*) show significantly different regulatory structures.

**Fig 1:**
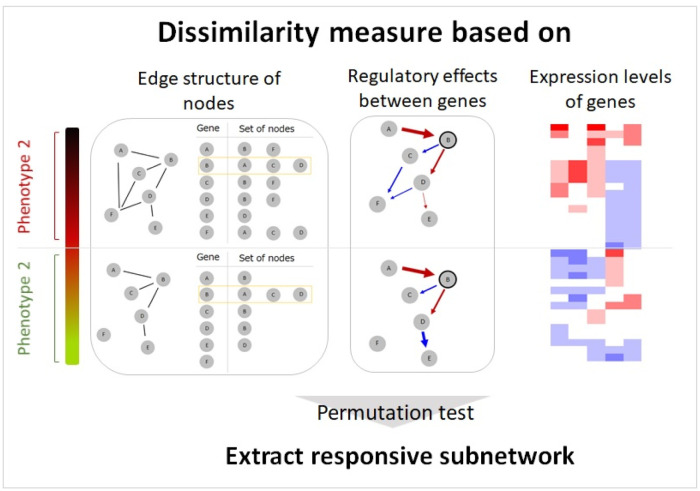
Overall framework of the proposed CIdrgn.

The algorithm for CIdrgn is given in **Algorithm**1. For the dissimilarity measure CIdrgn.2, similar procedures were applied to compute the permutation p.value_CIdrgn.2_ and identify the responsive subnetworks. The proposed statistic is based on comprehensive information of the gene regulatory network, that is, the dissimilarity of node edge structures, regulatory effects, and expression levels; thus, we can effectively extract biological condition-specific gene networks. Fig 1 shows overall framework of the proposed CIdrgn.

**Algorithm 1** Algorithm for CIdrgn

1: Construct gene regulatory networks for phenotypes A and B: *G*(*A*), *G*(*B*).

2: Compute the statistics Γ, Λ and Γ_Λ_.

3: Construct permutation samples $S_{A}^{\left(pm\right)}$ and $S_{B}^{\left(pm\right)}$, estimate gene networks based on the permutation samples *G*^*pm*^(*A*) and *G*^*pm*^(*B*) and compute the statistics Γ^(*pm*)^, Λ^(*pm*)^, and $\Gamma_{\Lambda }^{\left(pm\right)}$ for *pm* = 1, …*T*.

4: Normalize **Γ**, **Λ**, **Γ**_Λ_ and then compute CIdrgn.1 and CIdrgn^(*pm*)^.1 for *pm* = 1, …, *pm*.

5: Compute permutation p-values of the statistic CIdrgn.1: p.value_CIdrgn.1_.

6: If p.value_CIdrgn.1_ < *κ*, then select the subnetwork as a differentially regulated gene networks, where *κ* is a threshold.

7: Output the list of subnetworks.

### CIdrgn for cell line characteristic-specific gene networks

We consider cell line characteristic-specific gene regulatory networks that can be estimated using the following varying coefficient model [9]:

$$\begin{array}{c} y_{\ell }=\sum_{j=1}^{p}\beta_{j\ell }\left(m_{\alpha }\right)\cdot x_{j}+\varepsilon_{\ell },\;\ell =1,\ldots ,q, \end{array}$$ (17)

where *β*_*jℓ*_(*m*_*α*_) is the varying coefficient of the *j*^*th*^ regulator gene ***x***_*j*_ on *ℓ*^*th*^ target gene ***y***_*ℓ*_ for the *α*^*th*^ target sample with modulator *M* = *m*_*α*_, such as the cancer characteristics of the cell lines. The varying coefficient model enables us to construct the gene regulatory networks under varying conditions of cell line characteristics *M* = *m*_*α*_. The network of the *α*^*th*^ cell line can be represented using a weighted graph *G*_*α*_ = (*V*, *E*_*α*_, *W*_*α*_), where *W*_*α*_ = (*w*_*αij*_) is the edge weight, which can be computed based on ${\hat{\beta }}_{ij}\left(m_{\alpha }\right)$ and ${\hat{\beta }}_{ji}\left(m_{\alpha }\right)$. *E*_*α*_ ∈ *V* × *V* is the set of edges, where (*i*, *j*) ∈ *E*_*α*_ indicates the link between vertices *i* and *j* in the network of the *α*^*th*^ cell line.

We also propose the method for identifying responsive subnetwork from cell line characteristic-specific gene networks.

- Dissimilarity based on the regulatory effect of the sample-specific gene network
  For the sample-specific gene network, the regulatory effect of the *j*^*th*^ regulator gene on *ℓ*^*th*^ target gene in the *α*^*th*^ cell line is given as follows:
  $$\begin{array}{c} r_{\alpha \ell j}={\hat{\beta }}_{\ell j}\left(m_{\alpha }\right)\cdot x_{\alpha j},\;\text{for}\;j=1,\ldots ,p,\;\ell =1,\ldots ,q, \end{array}$$ (18)
  where *x*_*αj*_ is an expression level value of the *j*^*th*^ gene in the *α*^*th*^ cell line. We define the regulatory effect of phenotypes A and B as follows:
  $$\begin{array}{c} r_{\ell j}^{ss}\left(A\right)=\text{median}\left(r_{\alpha lj};\alpha \in A\right)\;\text{and}\;r_{\ell j}^{ss}\left(B\right)=\text{median}\left(r_{\alpha lj};\alpha \in B\right), \end{array}$$ (19)
  where A and B are the set of cell lines of phenotypes A and B, respectively. Let $R^{ss}\left(A\right)={\left\{r_{1j}^{ss}\left(A\right),\ldots ,r_{qj}\left(A\right),r_{j1}^{ss}\left(A\right),\ldots ,r_{jp}\left(A\right)\right\}}^{T}$ and $R^{ss}\left(B\right)={\left\{r_{1j}^{ss}\left(B\right),\ldots ,r_{qj}\left(B\right),r_{j1}^{ss}\left(B\right),\ldots ,r_{jp}\left(B\right)\right\}}^{T}$ be regulatory effect vectors of the *j*^*th*^ gene in the network of *α*^*th*^ cell line for phenotypes A and B, respectively. We consider the statistic to measure the dissimilarity of the regulatory effect of the *j*^*th*^ gene in sample-specific gene networks as follows
  $$\begin{array}{c} \gamma_{j}^{ss}\left(A,B\right)=\frac{1}{q+p}\left|\right|R^{ss}\left(A\right)-R^{ss}\left(B\right){\left|\right|}_{2}^{2}. \end{array}$$ (20)
  We then propose the following statistic to measure dissimilarity of the regulatory effects of genes in a subnetwork *G*_*α*_,
  $$\begin{array}{c} \Gamma^{ss}=\frac{1}{\left|V\right|}\sum_{j\in V}\Gamma_{j}^{ss}\left(A,B\right). \end{array}$$ (21)
- Dissimilarity of the node edge structures for sample-specific gene network
  We consider $\cup_{\alpha \in A}N_{\alpha j}$ and $\cup_{\alpha \in B}N_{\alpha j}$ as the sets of edges of the *j*^*th*^ gene in phenotypes A and B, respectively. We then define the following node similarity:
  $$\begin{array}{c} N_{j}^{ss}\left(A,B\right)=\frac{|\left(\cup_{\alpha \in A}N_{\alpha j}\right)\cap \left(\cup_{\alpha \in B}N_{\alpha j}\right)|}{|\left(\cup_{\alpha \in A}N_{\alpha j}\right)\cup \left(\cup_{\alpha \in B}N_{\alpha j}\right)|}, \end{array}$$ (22)
  where *N*_*αj*_ is the sets of nodes that are connected to the *j*^*th*^ gene in the network of the *α*^*th*^ cell line. The Jaccard distance of the *j*^*th*^ node in the cell line characteristic-specific gene network is given as follows:
  $$\begin{array}{c} \lambda_{j}^{ss}\left(A,B\right)=1-N_{j}^{ss}\left(A,B\right). \end{array}$$ (23)
  Similar to (10), the dissimilarity measure of the edge structure is given as follows:
  $$\begin{array}{c} \Lambda^{ss}=\frac{1}{\left|V\right|}\sum_{j\in V}\lambda_{j}^{ss}\left(A,B\right). \end{array}$$ (24)

We then proposed the following statistics to identify differentially regulated gene networks in the cell line characteristic-specific gene network analysis.

$$\begin{array}{cc} & {\text{CIdrgn}}^{ss}.1=\Gamma_{\Lambda }^{ss}+D_{\text{SAM}-\text{GS}}, \end{array}$$ (25)

$$\begin{array}{cc} & {\text{CIdrgn}}^{ss}.2=\Gamma^{ss}+\Lambda^{ss}+D_{\text{SAM}-\text{GS}}, \end{array}$$ (26)

where

$$\begin{array}{c} \Gamma_{\Lambda }^{ss}=\frac{1}{\left|V\right|}\sum_{j\in V}\{\gamma_{j}^{ss}\left(A,B\right)+\gamma_{j}^{ss}\left(A,B\right)\cdot \lambda_{j}^{ss}\left(A,B\right)\}. \end{array}$$ (27)

For the cell line characteristic-specific gene network, the condition-responsive subnetworks are extracted based on the permutation p-value of CIdrgn^*ss*^.1 and CIdrgn^*ss*^.2, which are consistent with p.value_CIdrgn.1_ and p.value_CIdrgn.2_.

## Monte Carlo simulations

**Fig 2:**
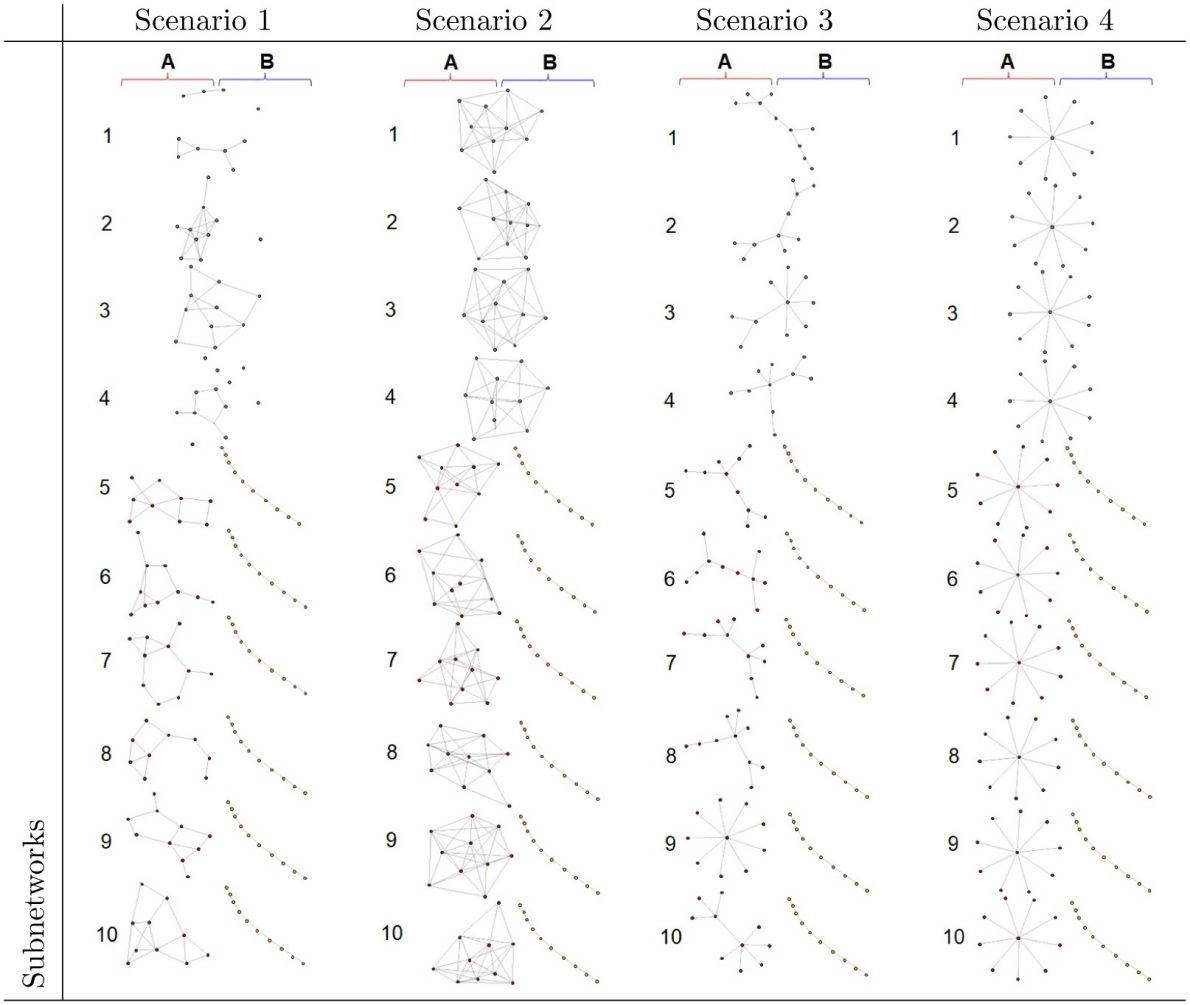
Subnetwork structures.

Monte Carlo simulations were conducted to investigate the performance of the proposed CIdrgn. Suppose that we have two phenotypes A and B and 10 subnetworks consisting of 10 genes, where the sample sizes of phenotypes A and B are *n*_*A*_ = 50 and *n*_*B*_ = 50, respectively. We consider four common subnetworks of the two phenotypes A and B, six A-specific and six B-specific subnetworks. The expression levels of the *p* genes in 100 (= *n*_*A*_ + *n*_*B*_) cell lines were generated using the following procedure. In scenario 1, we generated four precision matrices $\Omega_{c}^{nw}$ for *nw* = 1,.., 4 common subnetworks from “random’’ graph structure using *huge.generator* in R package Huge, where the off-diagonal elements of the precision matrix were set to 0.5 and the positive number 0.2 was added to the diagonal elements of the precision matrix, which is consistent with [10]. For each 10 genes consisting of the 4 common subnetworks, the expression levels of 100 cell lines were generated from $N(0_{10},\Omega_{c}^{-1})$. For the six A-specific and B-specific subnetworks, the precision matrices ($\Omega_{A}^{nw}$ and $\Omega_{B}^{nw}$ for *nw* = 1, …, 6) were generated from the “random” and “band” graph structures, respectively. For each 10 genes consisting of the each six A- and six B-specific subnetworks, we generated expression levels of *n*_*A*_ = *n*_*B*_ = 50 cell lines from $N(0_{10},\Omega_{A}^{-1})$ and $N(\mu_{10},\Omega_{B}^{-1})$, respectively. The expression levels of the remaining *p* − 100 genes were generated from *N*(**0**_*p*−100_, ***I***_*p*_). Scenarios 2, 3, and 4 were similar to scenario 1 except that the precision matrices $\Omega_{c}^{nw}$ for *nw* = 1,.., 4 and $\Omega_{A}^{nw}$ for *nw* = 1, …, 6 have “cluster”, “scale-free”, and “hub” graph structures for scenarios 2, 3, and 4, respectively. Fig 2 shows the graph structures of the common subnetworks and A- and B-specific subnetworks.

We considered *p* = 1000 and ***μ***_10_ = (0.3, 0.3, …, 0.3)^*T*^, and the gene regulatory networks were estimated using the lasso method [11], where the response and predictor variables are the expression levels of the target gene and regulator genes, respectively. We evaluated the proposed CIdrgn by comparing it with the statistics SAM-GS and GSCA. The permutation p-values of the statistics SAM-GS and GSCA were computed, and responsive subnetworks were extracted by the permutation p-values with a significance level of 0.05, such as in the case of CIdrgn.

The recall, precision, true negative rate (TNR), F-measure, and accuracy results are given in the column labeled “subnetwork size:10” of Table 1. CIdrgn provides effective results for responsive subnetwork identification overall, although limited differences are observed. In particular, CIdrgn.2 shows effective results in terms of precision, true negative rate, F-measure, and accuracy. The methods show similar results for the true positive rate (i.e., Recall) for six responsive network identifications, while our method provides outstanding performance for the true negative rate (TNR) for four common subnetworks.

**Table 1:** Simulation results.

	Methods	Subnetwork size: 10				Subnetwork size: 50			
		SCN.1	SCN.2	SCN.3	SCN.4	SCN.1	SCN.2	SCN.3	SCN.4
Recall	SAMGS	0.970	0963	0.950	0.933	1.000	1.000	1.000	1.000
	GSCA	**1.000**	**1.000**	**0.993**	**1.000**	1.000	1.000	1.000	1.000
	CIdrgn.1	**1.000**	0.997	**0.993**	0.997	1.000	1.000	1.000	1.000
	CIdrgn.2	0.993	0.993	0.990	0.997	1.000	1.000	1.000	1.000
Precision	SAMGS	0.986	0.988	0.982	0.985	0.991	0.986	0.974	0.961
	GSCA	0.966	0.970	0.954	0.969	0.961	0.971	0.972	0.958
	CIdrgn.1	**0.989**	0.994	0.984	**0.991**	**0.994**	0.989	**0.991**	0.969
	CIdrgn.2	**0.989**	**0.997**	**0.989**	**0.991**	**0.994**	**0.991**	**0.994**	**0.970**
TNR	SAMGS	0.975	0.980	0.970	0.975	0.985	0.975	0.955	0.930
	GSCA	0.940	0.945	0.915	0.945	0.930	0.950	0.950	0.925
	CIdrgn.1	**0.980**	0.990	0.970	0.985	**0.990**	0.980	**0.985**	**0.945**
	CIdrgn.2	**0.980**	**0.995**	**0.980**	**0.985**	**0.990**	**0.985**	**0.990**	**0.945**
F.measure	SAMGS	0.975	0.974	0.962	0.954	0.995	0.992	0.986	0.979
	GSCA	0.982	0.984	0.971	0.983	0.979	0.985	0.985	0.977
	CIdrgn.1	**0.994**	**0.995**	0.987	**0.994**	**0.997**	0.994	0.995	0.983
	CIdrgn.2	0.990	**0.995**	**0.988**	**0.994**	**0.997**	**0.995**	**0.997**	**0.984**
Accuracy	SAMGS	0.972	0.970	0.958	0.950	0.994	0.990	0.982	0.972
	GSCA	0.976	0.978	0.962	0.978	0.972	0.980	0.980	0.970
	CIdrgn.1	**0.992**	**0.994**	0.984	**0.992**	**0.996**	0.992	0.994	**0.978**
	CIdrgn.2	0.988	**0.994**	**0.986**	**0.992**	**0.996**	**0.994**	**0.996**	**0.978**

We also consider the scenarios for a large-scale subnetwork, that is, each 10 subnetworks consist of 50 genes. The results for the large-scale subnetwork scenarios are presented in the column labeled “subnetwork size:50” in Table 1. The results show that our strategy also provides outstanding performance for large-scale subnetwork situations, where CIdrgn.2 also shows the most effective results for differentially regulated gene network identification.

### Monte Carlo simulations: Sample-specific gene network analysis

For the cell line characteristic-specific gene regulatory networks, we considered the same network structures given in Fig 2. For sample-specific gene regulatory strength, we generated precision matrices as follows:

$$\begin{array}{cc} & \Omega_{ij}^{\alpha }\left(A\right)=\Omega_{ij}\left(A\right)\times 0.5+\left\{\Omega_{ij}\left(A\right)-\Omega_{ij}\left(A\right)\times 0.5\right\}\delta_{\alpha },\;\alpha =1,\ldots ,50,\\ & \Omega_{ij}^{\alpha }\left(B\right)=\Omega_{ij}\left(B\right)\times 0.5+\left\{\Omega_{ij}\left(N\right)-\Omega_{ij}\left(B\right)\times 0.5\right\}\delta_{\alpha },\;\alpha =1,\ldots ,50. \end{array}$$ (28)

The gene expression levels were determined using the same method applied in the gene network analysis with bulk cell lines except that the expression of genes in six A-specific and six B-specific subnetworks was generated as ***x***_*α*_(*A*) ∼ *N*(**0**_10_, **Ω**^*α*^(*A*)) and ***x***_*α*_(*B*) ∼ *N*(***μ***_10_, **Ω**^*α*^(*B*)) for *α* = 1, …, 50. The modulator *M* = (*m*_1_, …, *m*_100_)^*T*^ that describes the characteristics of cell lines was generated from the uniform distribution *U*(−1, 1).m The cell line characteristic-specific gene network was estimated using SiGN-L1 (https://sign.hgc.jp/signl1/) [7].

Table 2 shows the results for cell line characteristic-specific gene networks.

**Table 2:** Simulation results: Cell line characteristic analysis.

	Methods	Subnetwork size: 10				Subnetwork size: 50			
		SCN.1	SCN.2	SCN.3	SCN.4	SCN.1	SCN.2	SCN.3	SCN.4
Recall	SAMGS	1.000	1.000	1.000	1.000	1.000	1.000	1.000	1.000
	GSCA	0.990	0.993	0.980	1.000	1.000	1.000	1.000	1.000
	CIdrgn.1	1.000	1.000	1.000	1.000	1.000	1.000	1.000	1.000
	CIdrgn.2	1.000	1.000	1.000	1.000	1.000	1.000	1.000	1.000
Precision	SAMGS	0.959	0.966	**0.980**	0.964	0.976	0.956	0.969	0.986
	GSCA	0.966	0.962	0.966	0.961	0.964	0.963	0.967	0.961
	CIdrgn.1	0.980	**0.983**	0.974	**0.969**	0.978	**0.969**	0.980	**0.994**
	CIdrgn.2	**0.986**	0.979	**0.980**	0.966	**0.979**	0.955	**0.986**	0.989
TNR	SAMGS	0.925	0.940	**0.965**	0.935	0.955	0.920	0.945	0.975
	GSCA	0.940	0.930	0.940	0.930	0.935	0.935	0.940	0.930
	CIdrgn.1	0.965	**0.970**	0.955	**0.945**	**0.960**	**0.945**	0.965	**0.990**
	CIdrgn.2	**0.975**	0.960	**0.965**	0.940	**0.960**	0.920	**0.975**	0.980
F.measure	SAMGS	0.977	0.982	**0.989**	0.980	0.987	0.976	0.983	0.992
	GSCA	0.976	0.975	0.971	0.979	0.980	0.980	0.982	0.979
	CIdrgn.1	0.989	**0.991**	0.986	**0.983**	**0.988**	**0.983**	0.989	**0.997**
	CIdrgn.2	**0.992**	0.988	**0.989**	0.982	**0.988**	0.976	**0.992**	0.994
Accuracy	SAMGS	0.970	0.976	**0.986**	0.974	0.982	0.968	0.978	0.990
	GSCA	0.970	0.968	0.964	0.972	0.974	0.974	0.976	0.972
	CIdrgn.1	0.986	**0.988**	0.982	**0.978**	**0.984**	**0.978**	0.986	**0.996**
	CIdrgn.2	**0.990**	0.984	**0.986**	0.976	**0.984**	0.968	**0.990**	0.992

CIdrgn provides effective results for condition-responsive subnetwork identification in cell line characteristic-specific gene network analyses. CIdrgn.1 outperforms in scenarios 2 and 4, while CIdrgn.2 shows an effective performance for scenarios 1 and 3.

In short, our proposed strategy provides effective results for differentially regulated gene network identification in bulk cell line gene networks and cell line characteristic-specific gene networks. This finding implies that the incorporation of comprehensive information on gene regulatory systems is crucial for differential gene regulatory network analyses.

## Gastric cancer and XELOX-responsive gene regulatory network analysis

Gastric cancer (GC) is the one of the leading causes of cancer-related death worldwide. Although chemotherapy represents the standard treatment for GC, it is not always effective because of drug resistance in cancer cell lines. Uncovering crucial markers and their regulatory networks that play key roles in the mechanisms of acquired drug resistance are a vital issue to enhance chemotherapy efficacy of gastric cancer.

We applied the proposed strategy to identify GC and chemotherapy -responsive gene regulatory networks. We used a publicly available large-scale dataset from the Cancer Dependency Map (DepMap) (https://depmap.org/portal/), where the RNA expression of 19,221 genes and 1,406 cell lines from the CCLE dataset (22Q2) and the drug sensitivity of 4,686 compounds and 578 cell lines from the PRISM repurposing primary screen can be downloaded. Because CIdrgn.2 and CIdrgn^*ss*^.2 show outstanding performance in simulation studies, we use CIdrgn.2 and CIdrgn^*ss*^.2 for GC and XELOX-responsive gene regulatory network analysis.

### GC-responsive gene regulatory network identification

To identify the GC-responsive gene regulatory networks, we considered 41 GC and 1365 non-gastric cancer (NGC) cell lines as the two phenotypes and then estimated GC gene regulatory networks based on the GC cell lines. The NGC gene network was estimated using 41 randomly selected cell lines from 1365 NGC cell lines, and the construction process was repeated 50 times based on 41 randomly selected NGC cell lines. The permutation GC and NGC networks were also estimated using the 41 cell lines randomly selected from among the 1406 cell lines, and the permutation NGC network construction process was also repeated 50 times. We then extracted subnetworks from the estimated GC and NGC networks and permutation GC and NGC networks. For NGC networks, only subnetworks existing in all 50 iterations were considered as NGC networks (permutation NGC networks). The permutation of network estimation was repeated 500 times. We extracted edges with a size greater than 0.7 and identified the GC/NGS-responsive network based on a permutation p-value with a significance level of 0.01. A total of 106 differentially regulated subnetworks were identified, where the identified 106 sub-networks are gastric cancer cell line specific gene networks, i.e., the sub-networks are not existed in NGC gene networks.

**Fig 3:**
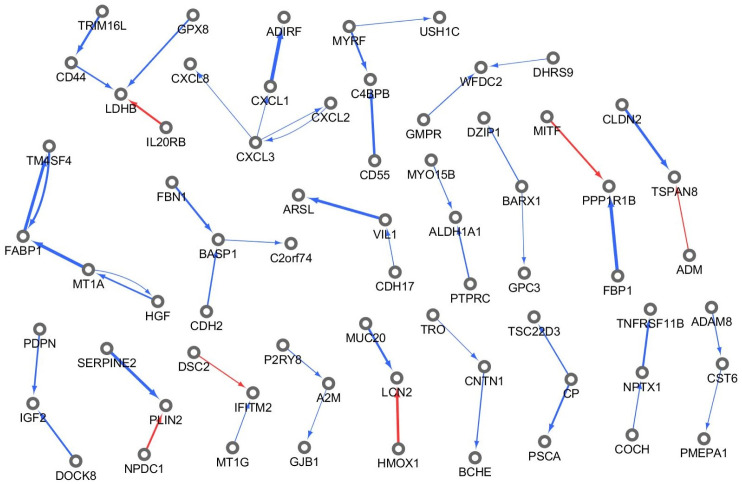
Differentially regulated gene network of GC.

To verify the efficiency of the method, we visualized the identified GC-specific gene regulatory networks based on subnetworks with a size greater than 3 (i.e., a subnetwork with more than three nodes), as shown in Fig 3. The chemokine (C-X-C motif) ligand (CXCL) family (i.e., CXCL1, CXCL2, CXCL3 and CXCL8) is identified as a GC-responsive gene regulatory system. Moreover, the CDH gene family (CDH2 and CDH17) has GC-responsive regulatory structures. The identified GC-responsive markers (i.e., CXCL and CDH gene families) have been studied as GC markers, and their involvement in mechanisms related to GC have been demonstrated in many studies.

#### CXCL family

The CXCL family plays an important role in the control of inflammation and angiogenesis because their specific receptors are expressed by both leukocytes and endothelial cells [12]. Chen et al. [13] revealed that the CXCL family represents markers for the development of GC and demonstrated that it is involved in the pathogenesis of GC. In particular, the mRNA expression levels of the CXCL family have been identified as promising prognostic indicators of Epstein-Barr virus-associated GC [14]. Zhou et al. [15] revealed that the CXCL10/CXCR3 axis promotes the invasion of GC via the PI3K/AKT pathway-dependent matrix metalloproteinase production.

#### CDH gene family

The CDH gene family has been identified as a marker of tissue morphogenesis, sorting, and cell adhesion, which are important processes for tissue formation and maintenance.

CDH2, encoded by N-cadherin, is involved in the transformation of human GC cells and associated with intracellular events of gastric carcinogenesis and high expression of corresponding transcription factors [16]. GC cell invasion is upregulated by Twist and accompanied by CDH2 [17]. GC invasiveness is inhibited by microRNA-145-5p, which targets CDH2 to suppress epithelial-mesenchymal transition (EMT) [18]. Jiang et al. [18] revealed that CDH2 is involved in the molecular mechanisms that regulate GC cell invasion and migration, especially EMT progression [19]. Aberrant CDH2 expression is involved in the invasion and metastasis of GC [20].

The expression of CDH17 is increased in GC, and its silencing suppresses the metastasis and proliferation of GC cells [21]. CDH17 has been identified as a useful target molecule, and its crucial roles in the comprehensive detection and localization of GC metastases have been revealed earlier [22]. Mutation of E-cadherin (CDH1) represents the most common germline mutation in GC [23]. In particular, mutations in CDH1 lead to hereditary diffuse GC [23, 24].

**Fig 4:**
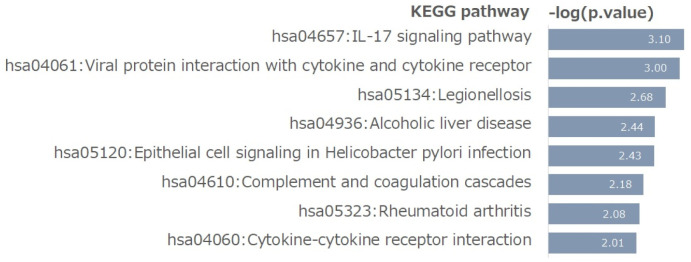
Kyoto Encyclopedia of Genes and Genomes (KEGG) analysis.

To identify the biological processes involved in the identified GC-responsive subnetworks, we performed a Kyoto Encyclopedia of Genes and Genome (KEGG) pathway enrichment analysis of the GC-responsive markers. Fig 4 shows the enriched pathways corresponding to the p-value (i.e., -log(p.value)). As shown in Fig 4, the most enriched pathway is the “IL-17 signaling pathway” grouping of the CXCL family (i.e., CXCL8, LCN2, CXCL1, CXCL3, and CXCL2). Furthermore, the “viral protein interaction with cytokine and cytokine receptor” and “cytokine-cytokine receptor interaction” pathways were also enriched for GC-responsive markers. The pathway “epithelial cell signaling in Helicobacter pylori infection” was also identified as an enriched pathway. These findings imply that GC-responsive networks may be involved in EMT-related mechanisms of GC.

### XELOX-responsive gene regulatory network identification

We also performed a GC chemotherapy-responsive gene regulatory network identification. We focused on the anticancer drugs capecitabine and oxaliplatin, which have been used as standard treatments for gastric and colorectal cancer, and their combination is known as the XELOX regimen. For XELOX, we extracted cell lines that had non-missing values in sensitivities to both capecitabine and oxaliplatin and matched the cell lines with the expression level dataset. In total, 520 cell lines were extracted. We then estimated 520 capecitabine- and capecitabine-sensitive gene networks using SiGN-L1 [7]. We considered the cell lines with the top 100 highest and lowest drug sensitivity values as drug-sensitive and drug-resistant cell lines, respectively. The sensitive and resistant cell lines for both capecitabine and oxaliplatin were defined as XELOX-sensitive and XELOX-resistant cell lines, and 18 XELOX-sensitive and 24 XELOX-resistant cell lines were considered. From the 520 capecitabine-sensitive gene networks, we extracted 18 and 24 networks corresponding to the 18 and 24 XELOX-sensitive and XELOX-resistant cell lines, respectively. The 18 sensitive and 24 resistance permutated networks were also randomly selected from 520 capecitabine-sensitive gene networks. By using the permutation p-value of CIdrgn^*ss*^.2 with a significance level of 0.01, we identified 62 capecitabine-responsive subnetworks. For oxaliplatin, similar processes were performed, and nine oxaliplatin-responsive subnetworks were identified. Among the genes in the 62 capecitabine- and 9 oxaliplatin-responsive subnetworks, the common genes in the sensitive (resistant) networks were defined as XELOX-sensitive (resistant) markers. We then extracted edges of the XELOX-sensitive (resistance) markers from the capecitabine and oxaliplatin-sensitive (resistance) gene networks and constructed XELOX-sensitive (resistant) networks, where the edge size was given as an average value from the 18 XELOX-sensitive and 24 XELOX-resistant networks.

**Fig 5:**
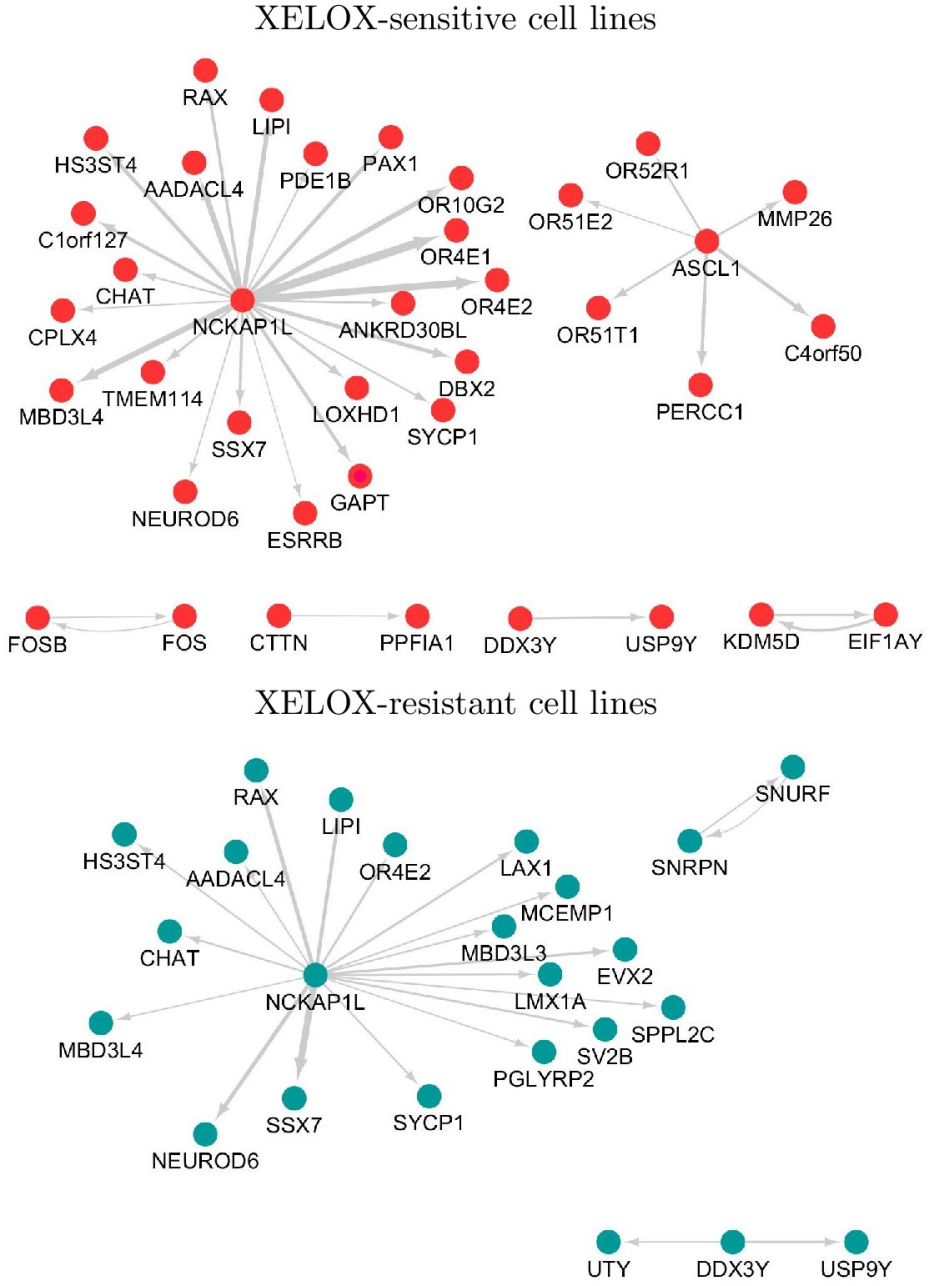
XELOX-responsive gene regulatory networks.

Fig 5 shows the XELOX-responsive gene regulatory networks for the XELOX-sensitive and XELOX-resistant cell lines. The networks of XELOX-sensitive and XELOX-resistant cell lines showed different gene regulatory structures despite presenting common genes, and their networks were observed for both sensitive and resistant cell lines. The subnetwork of NCKAP1L was the largest XELOX-responsive gene regulatory network. The regulatory structure between NCKAP1L/ASCL1 and the OR family can be considered a XELOX-sensitive gene regulatory system. ASCL1 is an important gene in XELOX-sensitive cell lines, whereas the sub-network disappears from sensitive to resistance cell lines. Furthermore, the subnetwork of the FOS gene family can also be considered a XELOX-sensitive network.

Table 3 shows the identified XELOX-responsive markers and their association with GC/colorectal cancer and chemotherapy.

**Table 3:** Identified markers and evidence.

Genes		Gastric cancer	Colorectal cancer	Chemotherapy
NCKAP1L			[27, 28]	immune checkpoint inhibitors [29],
				doxorubicin [30]
				cisplatin[31]
ASCL1		[32–34]	[32]	platinum [35], cabozantinib [36]
				Oxaliplatin [37]
				doxorubicin, octreotide [38]
Olfactory Receptor family	OR10G2	[39–41]	[39, 42, 43]	anthraquinone [44]
	OR4E1			
	OR4E2			
	OR51E2			
	OR51T1			
	OR52R1			
FOS gene family	FOS	[46]	[45, 47]	oxaliplatin [48, 49], eribulin[50]
	FOSB			carboplatin and paclitaxel [51]

#### NCKAP1L

The Nck-associated protein 1-like (NCKAP1L) gene is known as a DNA methylation-driven gene that promotes the malignancy of cancer cells [25, 26]. The expression of NCKAP1 is considered a marker of colon cancer [27]. NCKAP1 has been identified as a chemotherapy marker of immune checkpoint inhibitors [29]. NCKAP1 is associated with long-term DOX-induced cognitive dysfunction [30] Stark et al. [31] reported an association between NCKAP1 levels and cisplatin-induced apoptosis and cytotoxicity.

#### ASCL1

Expression levels of ASCL1 were identified as crucial markers of various GC subtypes, that is, lower expression of ASCL1 was observed in gastric mixed adenocarcinoma, gastrointestinal stromal tumors, and gastric intestinal-type adenocarcinoma [32]. In addition, overexpression of ASCL1 mRNA was observed in gastrointestinal carcinoid tumor cells compared with that in normal tissue [34]. ASCL1 was identified as a DEG between GC and normal tissue and an upregulated transcription factor in the gene network of the DEGs [33]. Furthermore, the downregulated expression of ASCL1 has been identified as a marker of GC and colon cancer [32]. Augustyn et al. [36] revealed that the ASCL1 pathway is a promising therapeutic target of cabozantinib for lung cancer. ASCL1 is involved in the anticancer mechanisms of doxorubicin and octreotide in neuroendocrine cancer [38].

#### OR family

OR family activation is a crucial marker for cancer cell growth and progression [39]. The OR family promotes the invasiveness and metastasis of colorectal cancer cells [43]. OR2B6 was detectable in colon and gastric carcinoma cell lines [39]. The long noncoding RNA of OR3A4 promotes metastasis and tumorigenicity in GC [40]. OR51B5 has been identified as a candidate oncogene in scirrhous-type GC [41].

Morita et al. [42] identified OR7C1 as a novel marker of colon cancer-initiating cells and a potent target of immunotherapy. Activation of the OR family members induces apoptosis and reduces cell migration and proliferation in colorectal cancer cells [39]. The OR family (i.e., OR1D2, OR4F15, and OR1A1) was also disrupted in colorectal cancer, and the importance of OR activity-associated genes in colorectal cancer was identified by Li et al. [43]. Choi et al. [44] found that the OR family is an important target for drug discovery and represents a crucial anticancer target.

#### FOS family (AP-1 transcription factor family)

The FOS gene family is involved in malignant transformation in several types of cancer, and its members (FOS, FOSB, FOSL1, and FOSL2) play crucial roles in cell proliferation and cancer development [45]. Abnormal expression of FOSB is a signature of tumor progression and poor survival. Overexpression of FOSB suppresses cell proliferation, clone formation, and migration, whereas silencing of FOSB leads to proliferation, clone formation, and migration in GC cell lines [46]. Tang et al. [46] suggested that FOSB could be a useful biomarker for the diagnosis and prognosis of GC. FOS family members have been identified as novel prognostic markers and therapeutic targets in colorectal cancer [45, 47]. The FOS family is involved in the antitumor efficacy of oxaliplatin, and the importance of the effect of oxaliplatin ‘on FOS signaling has been revealed [48, 49].

Dong et al. [50] demonstrated that upregulated FOS expression is a marker of low sensitivity to eribulin. Javellana et al. [51] revealed potentially clinically relevant roles of increased FOS family expression in driving the initial responses of tumors to carboplatin and paclitaxel and suggested that the FOS family will be a useful druggable target to counteract chemotherapy resistance.

**Fig 6:**
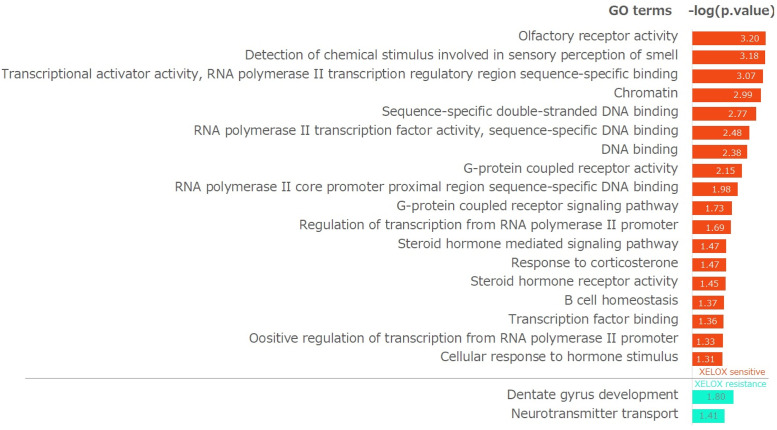
Gene Ontology (GO) pathway analysis of XELOX-sensitive (red) and XELOX-resistant (blue) specific markers.

For the XELOX-sensitive and XELOX-resistant subnetworks in Fig 5, we performed a Gene Ontology (GO) enrichment analysis. Fig 6 shows the significant GO terms corresponding to a p-value less than 0.01, where the enriched pathways of XELOX-sensitive and XELOX-resistant subnetworks are shown in red and blue, respectively. As shown in Fig 6, the XELOX-sensitive (red) and XELOX-resistant (blue) markers are enriched in different pathways, although the networks have common genes.

The most enriched pathway of the XELOX-sensitive markers is the “olfactory receptor activity” grouping of the OR family (i.e., OR4E2, OR4E1, OR10G2, OR52R1, OR51E2, OR51T1). Furthermore, the pathways related to “DNA binding”, “steroid hormone”, and “G protein-coupled receptor” were also identified as XELOX-sensitive gene markers. These findings imply that the XELOX-sensitive gene regulatory network is dominated by G protein-coupled receptors because the OR family belongs to the superfamily of G protein-coupled receptors [52]. For XELOX-resistance markers, the most enriched pathway is “neurotransmitter transport”, and it consists SV2B and CHAT, which have crucial molecular functions as drug targets [53].

Our results and those of previous studies show that the CXC family of chemokines may play a crucial role in the progression of GC. Furthermore, identifying the EMT-related mechanism involving the CDH gene family is essential for understanding the metastasis, proliferation, and development of GC and revealing the mechanism of the gene family in EMT-associated GC is vital to understanding the progression of GC. The GS- and anti-cancer drugs response- related mechanisms of ASCL1 that was identified as XELOX sensitive-specific hub gene were revealed in previous studies. Although its mechanisms of acquiring gastric cancer drug resistance have not yet been clearly demonstrated, our results and literatures suggest that the loss of activities and molecular interactions of the OR family with the ASCL1 and FOS families may represent a molecular mechanism underlying acquired drug resistance in GC cell lines.

## Conclusions

In this study, we introduce a novel strategy for differential gene regulatory network analysis. To identify condition-responsive gene regulatory networks describing biological condition-specific regulatory characteristics, we propose a computational method that measures the dissimilarity of subnetworks based on not only gene expression, but also regulatory effects between genes and edge structures. We also extended the proposed method to cell line characteristic-specific gene network analysis. Our method performs differential gene regulatory network analysis based on comprehensive information of the network structure; thus, we can effectively identify biological condition-responsive gene networks that cannot be detected using methods based on differences in gene expression levels. Monte Carlo simulations showed that our strategy demonstrates outstanding performance for responsive gene network identification in various network structure scenarios. It implies that incorporation of various network structures not only expression levels of genes is crucial to identify responsive networks.

We applied the proposed CIdrgn to the DepMap dataset and performed an analysis of GC and chemotherapy (XELOX) -responsive gene regulatory networks. Our strategy identified the molecular interplays of CXCL family (i.e., CXCL1, CXCL2, CXCL3 and CXCL8) and CDH gene family as GS responsive networks. For the XELOX-responsive gene networks, the regulatory structure between NCKAP1L/ASCL1 and the OR family are identified. The identified markers from the GC and XELOX-responsive network analysis provide strong evidence for the mechanisms related to GC, colorectal cancer, and various chemotherapies.

Our results suggest that the CXC family of chemokines and CDH genes may play a crucial role in GC tumor invasion, metastasis, and progression. Moreover, our findings indicate that revealing the mechanisms associated with genes in EMT-associated GC will be key to understanding GC progression. We also suggest that the loss of activity of the OR family with the ASCL1/FOS family may lead to drug resistance in cell lines, and inducers of this gene family can provide crucial information to improve the chemotherapy efficiency of XELOX.
